# Mesenchymal stem cell-derived exosomes protect trabecular meshwork from oxidative stress

**DOI:** 10.1038/s41598-021-94365-4

**Published:** 2021-07-21

**Authors:** Ying-chao Li, Juan Zheng, Xi-zi Wang, Xin Wang, Wen-jing Liu, Jian-lu Gao

**Affiliations:** 1 Department of Ophthalmology, Liaocheng People’s Hospital, Cheeloo College of Medicine, Shandong University, Liaocheng, 252000 Shandong China; 2 Department of Ophthalmology, Taian City Central Hospital, Taian, 271000 Shandong China; 3 Joint Laboratory for Translational Medicine Research, Beijing Institute of Genomics, Chinese Academy of Sciences & Liaocheng People’s Hospital, Liaocheng, 252000 Shandong China; 4 Department of Ophthalmology, Liaocheng People’s Hospital, Liaocheng, 252000 Shandong China

**Keywords:** RNA, Glaucoma, Gene regulation, RNA sequencing

## Abstract

This study aims to investigate the beneficial effects of exosomes derived from bone marrow mesenchymal stem cells (BMSCs) on trabecular meshwork cells under oxidative stress and predict candidate genes associated with this process. Trabecular meshwork cells were pretreated with BMSC-derived exosomes for 24 h, and exposed to 0.1 mM H_2_O_2_ for 6 h. Survival rate of trabecular meshwork cells was measured with CCK-8 assay. Production of intracellular reactive oxygen species (iROS) was measured using a flow cytometer. RT-PCR and ELISA were used to detect mRNA and protein levels of inflammatory cytokines and matrix metalloproteinases (MMPs). Sequencing of RNA and miRNA for trabecular meshwork cells from Exo and control groups was performed on BGISEQ500 platform. Phenotypically, pretreatment of BMSC-derived exosomes improves survival rate of trabecular meshwork cells exposed to H_2_O_2_, reduces production of iROS, and inhibits expression of inflammatory cytokines, whereas increases expression of MMPs. There were 23 miRNAs, 307 lncRNAs, and 367 mRNAs differentially expressed between Exo and control groups. Exosomes derived from BMSCs may protect trabecular meshwork cells from oxidative stress. Candidate genes responsible for beneficial effects, such as DIO2 and HMOX1, were predicted.

## Introduction

Glaucoma is a disease characterized by atrophy and depression of optic disc, visual field defect, and visual impairment. About 80 million people are estimated to be affected with glaucoma worldwide by 2020^1^. Based on status of iridocorneal angle, glaucoma can be classified into open-angle, closed-angle and developmental types, which are further divided into primary and secondary subtypes^2^. Notably, a major risk factor for primary open-angle glaucoma (POAG) is high intraocular pressure^3^. POAG is usually triggered by oxidative stress damage, mitochondrial dysfunction and abnormal accumulation of metabolites in trabecular meshwork cells, causing loss of cellular function^4^. As a result, it is of great significance to focus on protection of trabecular meshwork structure and function in POAG with elevated intraocular pressure.

Mesenchymal stem cells (MSCs) are self-replicating multipotent stromal cells isolated from mesenchymal tissues such as bone marrow^5^, umbilical cord blood^6^, adipose^7^ and dental pulp^8^. MSCs are proposed to play an important role in recovery and protection of tissues^9–11^, including nerve, bone, and cornea. Moreover, several studies reported potential ability of MSCs to recover or protect biological functions of trabecular meshwork. For instance, Roubeix et al.^12^ found that soluble factors secreted by MSCs might inhibit dysfunction of trabecular meshwork. In addition, Manuguerra et al.^13^ discovered that pro-recovery effects of MSCs on trabecular meshwork in a model of open-angle glaucoma are mediated in a paracrine manner rather than direct differentiation and repopulation of MSCs. Nevertheless, a study^9^ on protective effect of MSCs on injured retinas might have alluded exosome as the beneficial factor in question.

Exosomes, first reported in 1981^14^, comprise proteins, lipids, DNA, mRNA and microRNA^15^ surrounded by a phospholipid bi-layer secreted into extracellular space. It ranges from 30 to 150 nm in size^16–18^. Exosomal mRNAs and microRNAs may induce translation of new proteins after being transported to recipient cells via endocytosis^19,20^. MSCs have capability to mass synthesize and secrete exosomes^21^. Exosomes, as an important secreted constituent, might mediate and amplify restorative and protective functions of MSCs^22–24^. Therefore, this study speculates that the beneficial “factors” protecting trabecular meshwork cells in glaucoma models are exosomes secreted by MSCs. Exosomes secreted by bone marrow MSCs (BMSCs) contain over 150 different miRNA molecules^25^ that can be delivered to target cells. BMSC-derived exosomes may become critical tools for therapy of degenerative diseases^26^.

## Results

### Characterization of exosomes isolated from human BMSCs (hBMSCs)

**Figure 1:**
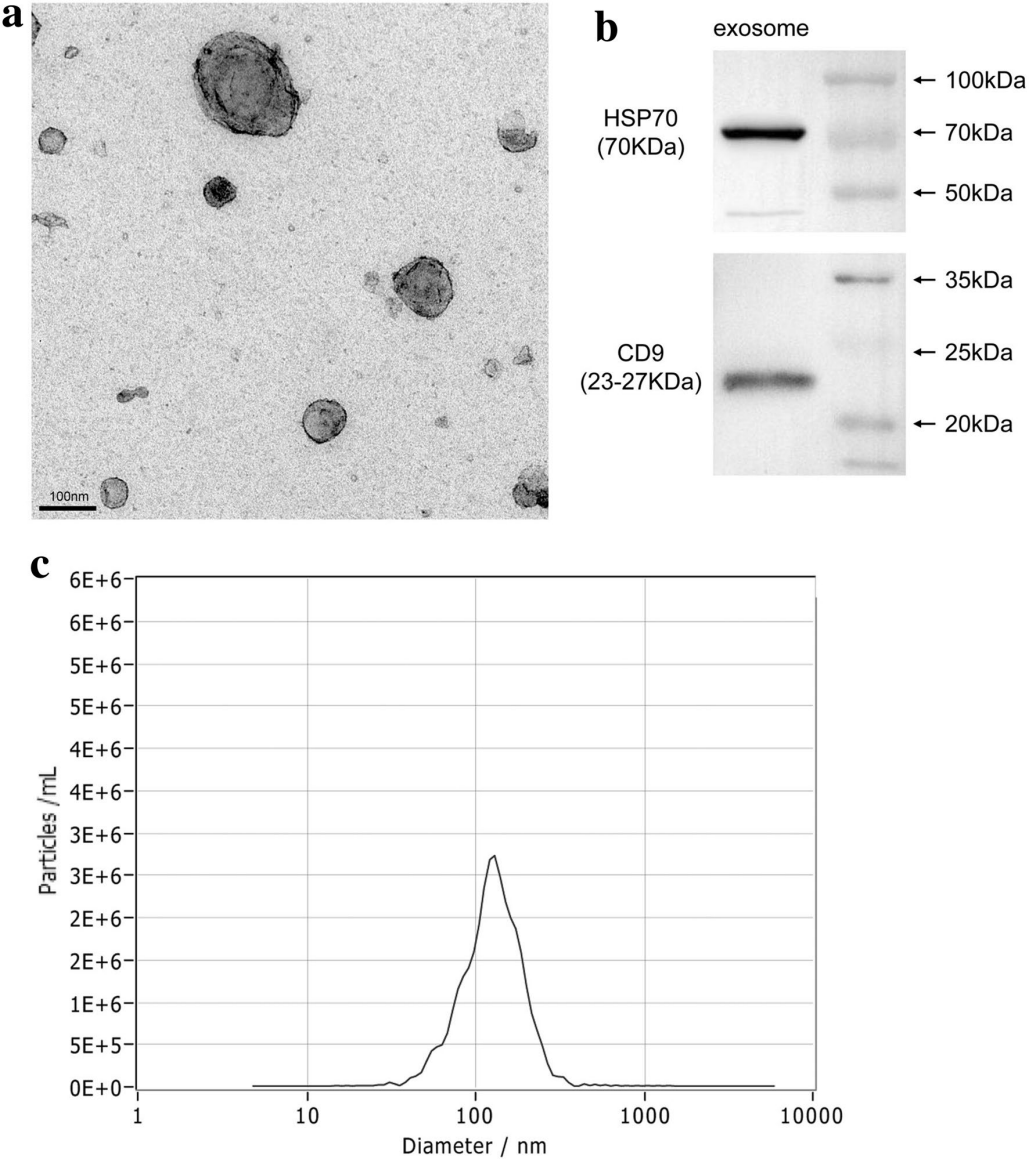
Identification of hBMSC-derived exosomes. (**a**) Identification of morphological characteristics of hBMSC-derived exosomes by transmission electron microscopy. The exosome concentrations were 1.5 to 1.6 × 10^9^/ml. (**b**) Western blot demonstrated characteristic markers of hBMSC-derived exosomes: HSP70 and CD9. (**c**) Size distribution of exosomes with a ZetaView analysis system.

Exosomes from hBMSCs were isolated using ultracentrifugation. Transmission electron microscopy revealed exosomes with a round shape and central depression (Fig. 1a). Based on Western blotting, exosomes expressed classic markers including HSP70 and CD9 (Fig. 1b). Using ZetaView, exosomes were evaluated to be a diameter of 130 nm in size (Fig. 1c).

### Exosomes isolated from hBMSCs are uptaken by human trabecular meshwork cells (hTMCs)

**Figure 2:**
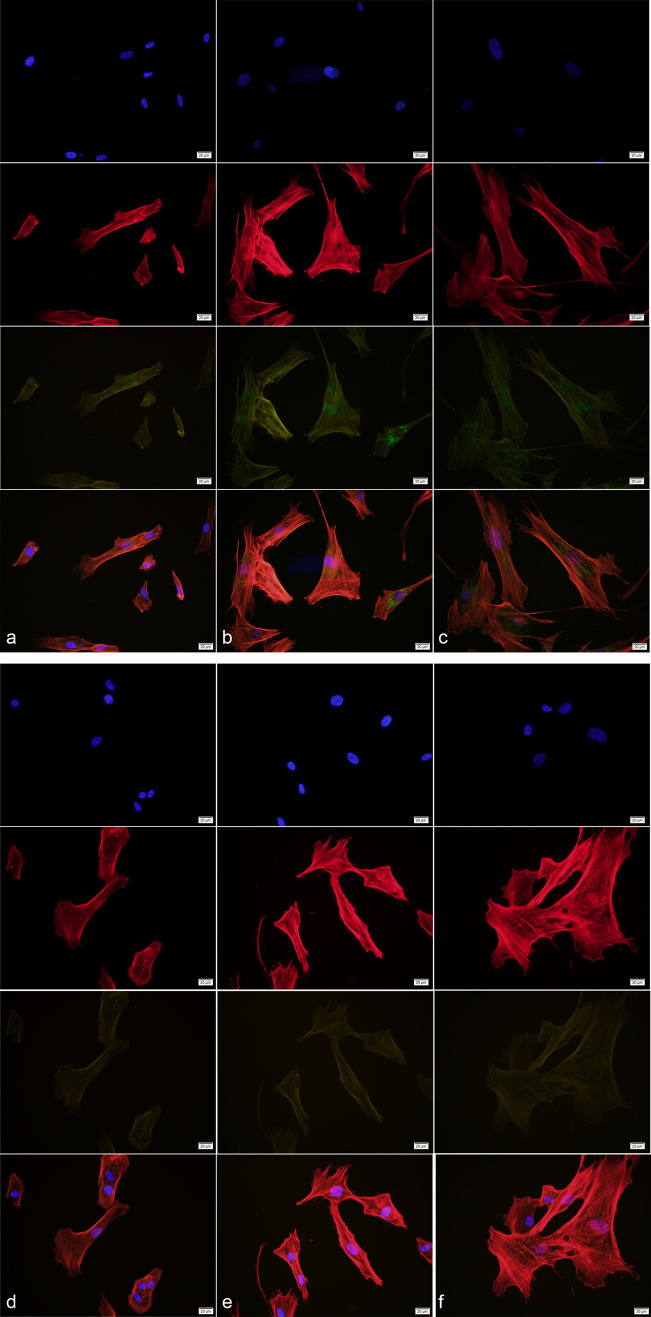
HTMCs uptake hBMSC-derived exosomes in different periods. HBMSC-derived exosomes were labeled with PKH67 (green), hTMCs nucleus were labeled with DAPI(blue), cytoplasm were labeled with phalloidine (red). HTMCs were incubated with hBMSC-derived exosomes for (**a**) 6 h, (**b**) 12 h, and (**c**) 24 h, respectively. Controls were incubated with dye only for (**d**) 6 h, (**e**) 12 h, and (**f**) 24 h, respectively.

The uptake of hBMSC-derived exosomes by hTMCs was observed at different time points i.e., 6, 12, and 24 h after incubation, respectively (Fig. 2). After incubation with labeled hBMSC-derived exosomes, green fluorescent particles in hTMCs were increased over time.

### HBMSC-derived exosomes improve viability of hTMCs exposed to H_2_O_2_

**Figure 3:**
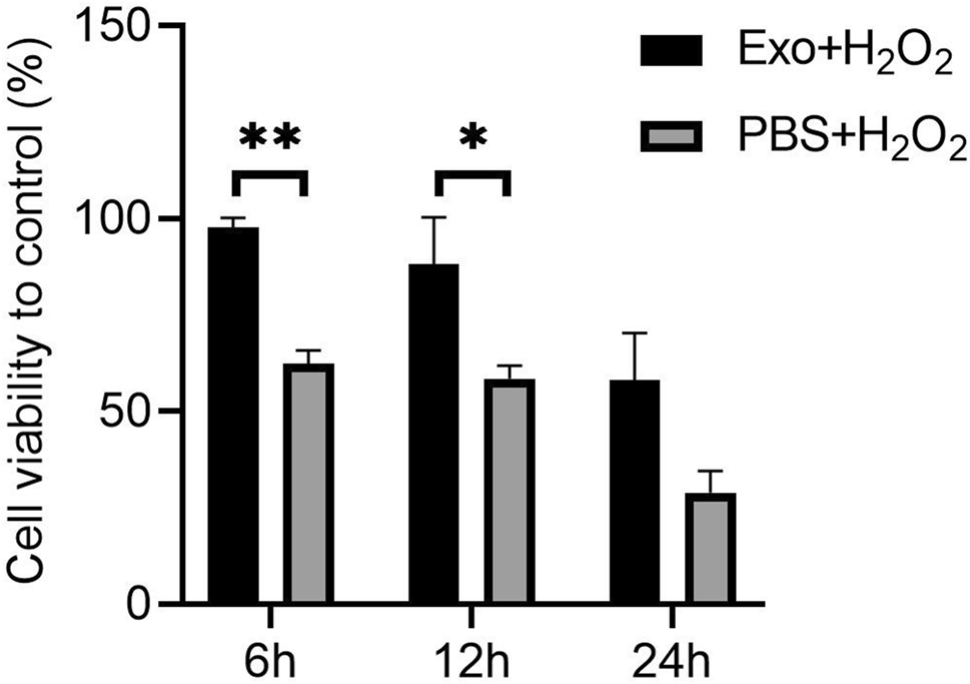
Pretreatment of hBMSC-derived exosomes improved viability of hTMCs exposed to H_2_O_2_. HTMCs were pretreated with hBMSC-derived exosomes or PBS for 24 h, then exposed to 0.1 mM H_2_O_2_ for different time points i.e., 6, 12, and 24 h, respectively. Cell viability was measured by CCK-8 assay. n = 3 for each condition. A P value was obtained by a two-tailed unpaired t-test. *P < 0.05 and **P < 0.01 compared to PBS + H_2_O_2_ group. Data were presented in Supplementary Table S2.

After 24 h of pretreatment with hBMSC-derived exosomes and washing 3 times with PBS, hTMCs were exposed to H_2_O_2_ (0.1 mM) for certain periods i.e., 6, 12, and 24 h, respectively. Notably, H_2_O_2_ (0.1 mM) significantly reduced survival rate of hTMC in a time-dependent manner. Meanwhile, pretreatment with hBMSC-derived exosomes improved viability of hTMCs exposed to 0.1 mM H_2_O_2_ (Fig. 3), and 6 h was chosen as the time point for the subsequent study.

### Effects of hBMSC-derived exosomes on production of intracellular reactive oxygen species (iROS) in H_2_O_2_-exposed hTMCs

**Figure 4:**
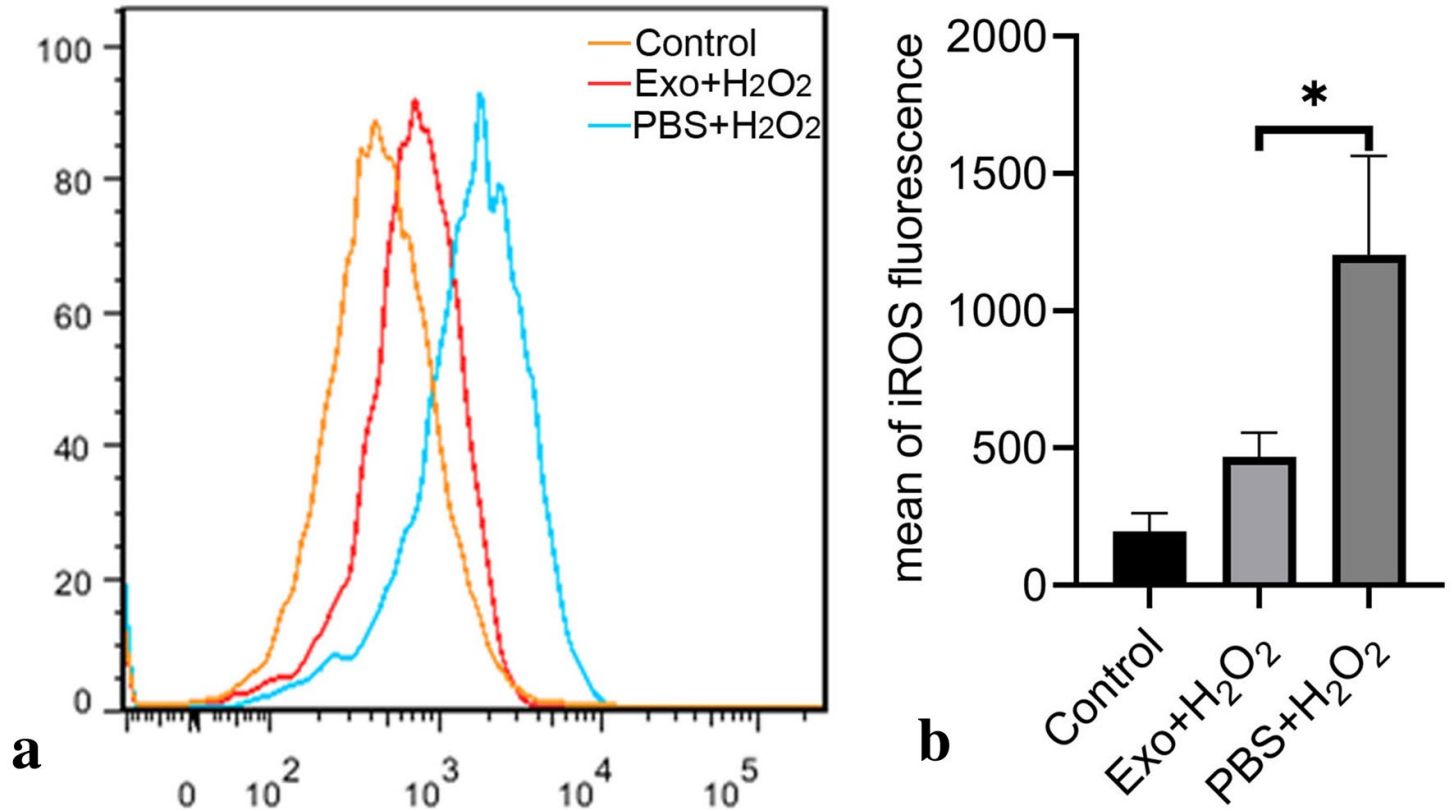
Pretreatment of hBMSC-derived exosomes reduced production of iROS in hTMCs when exposed to H_2_O_2_. HTMCs were pretreated with hBMSC-derived exosomes or PBS for 24 h, then exposed to H_2_O_2_ (0.1 mM) for 6 h, and a blank control group cultured with medium containing exosome-free serum. (**a**) Intracellular ROS was identified with DCFDA staining and measured by flow cytometry. (**b**) Quantification of iROS fluorescence. n = 3 for each condition. A P value was obtained by a two-tailed unpaired t-test. *P < 0.05 compared to PBS + H_2_O_2_ group. Data were presented in Supplementary Table S3.

After pretreatment with hBMSC-derived exosomes for 24 h and washing 3 times with PBS, hTMCs were exposed to H_2_O_2_ (0.1 mM) for 6 h. Exposure to H_2_O_2_ significantly induced a high amount of iROS production in hTMCs, whereas pretreatment with hBMSC-derived exosomes decreased production of iROS at 6 h, implying hBMSC-derived exosomes protected hTMCs against oxidative stress by reducing iROS (Fig. 4).

### Effects of hBMSC-derived exosomes on expression of proinflammatory factors in H_2_O_2_-exposed hTMCs

**Figure 5:**
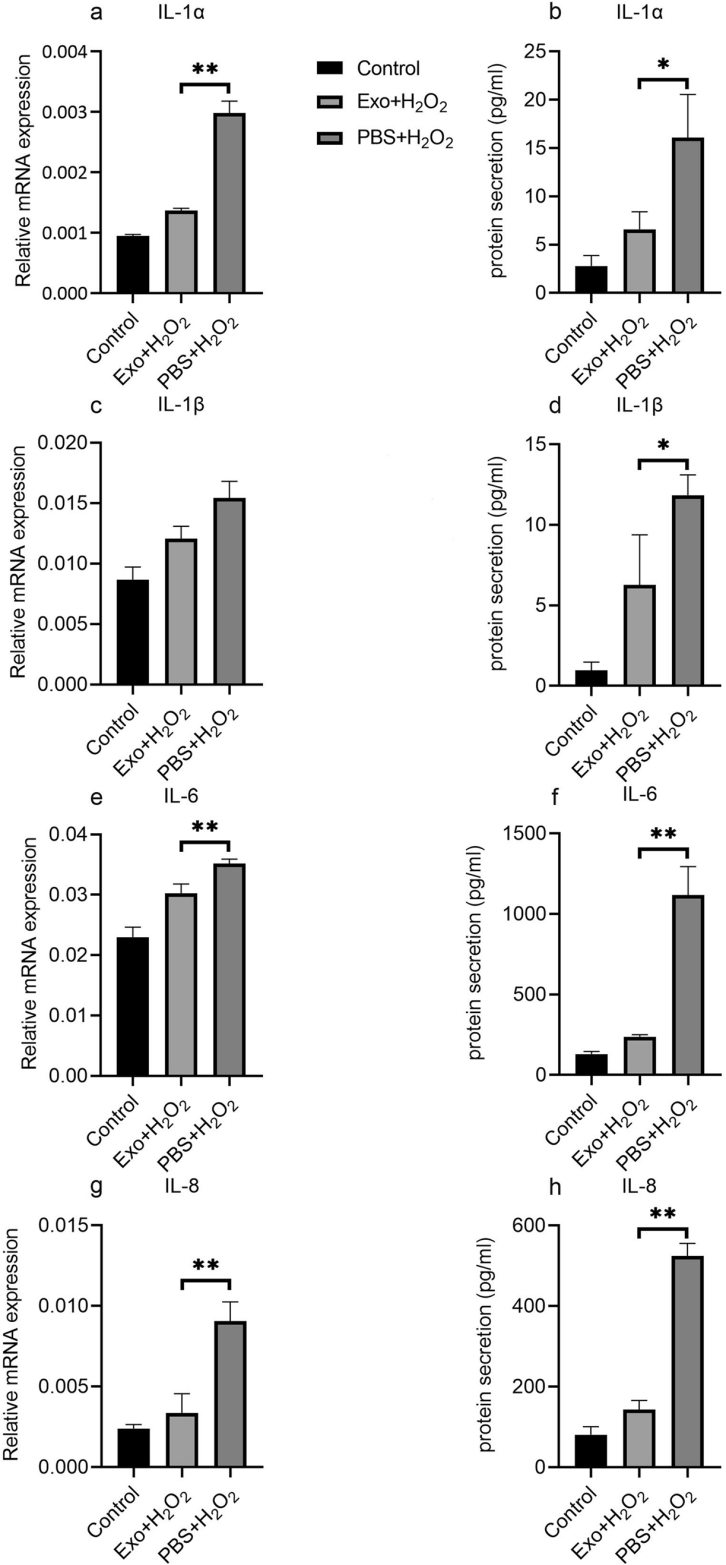
Pretreatment of hBMSC-derived exosome down-regulated the expression of inflammatory factors in hTMCs when exposed to H_2_O_2_. HTMCs were pretreated with hBMSC-derived exosomes or PBS for 24 h, then exposed to H_2_O_2_ (0.1 mM) for 6 h and a blank control group cultured with medium containing exosome-free serum. Then, hTMCs were assayed by RT-PCR to assess mRNA expression of IL-1α (**a**), IL-1β (**c**), IL-6 (**e**), and IL-8 (**g**). Gene expression levels were normalized to GAPDH and presented as relative ratios. Total hTMC supernatants were analyzed for IL-1α (**b**), IL-1β (**d**), IL-6 (**f**), and IL-8 (**h**). n = 3 for each condition. A P value was obtained by a two-tailed unpaired t-test. *P < 0.05; **P < 0.01 compared with PBS + H_2_O_2_ group. Data were presented in Supplementary Tables S4 and S5.

HTMCs were treated as described above. Expression levels of proinflammatory factors, including IL-1α, IL-1β, IL-6, and IL-8, were analyzed by RT-PCR and ELISA. High levels of IL-1α, IL-1β, IL-6, and IL-8 in hTMCs exposed to H_2_O_2_ were observed. Pretreatment with hBMSC-derived exosomes down-regulated IL-1α, IL-1β, IL-6, and IL-8 in hTMCs exposed to H_2_O_2_ (Fig. 5).

### Effects of hBMSC-derived exosomes on expression of MMPs in H_2_O_2_-exposed hTMCs

**Figure 6:**
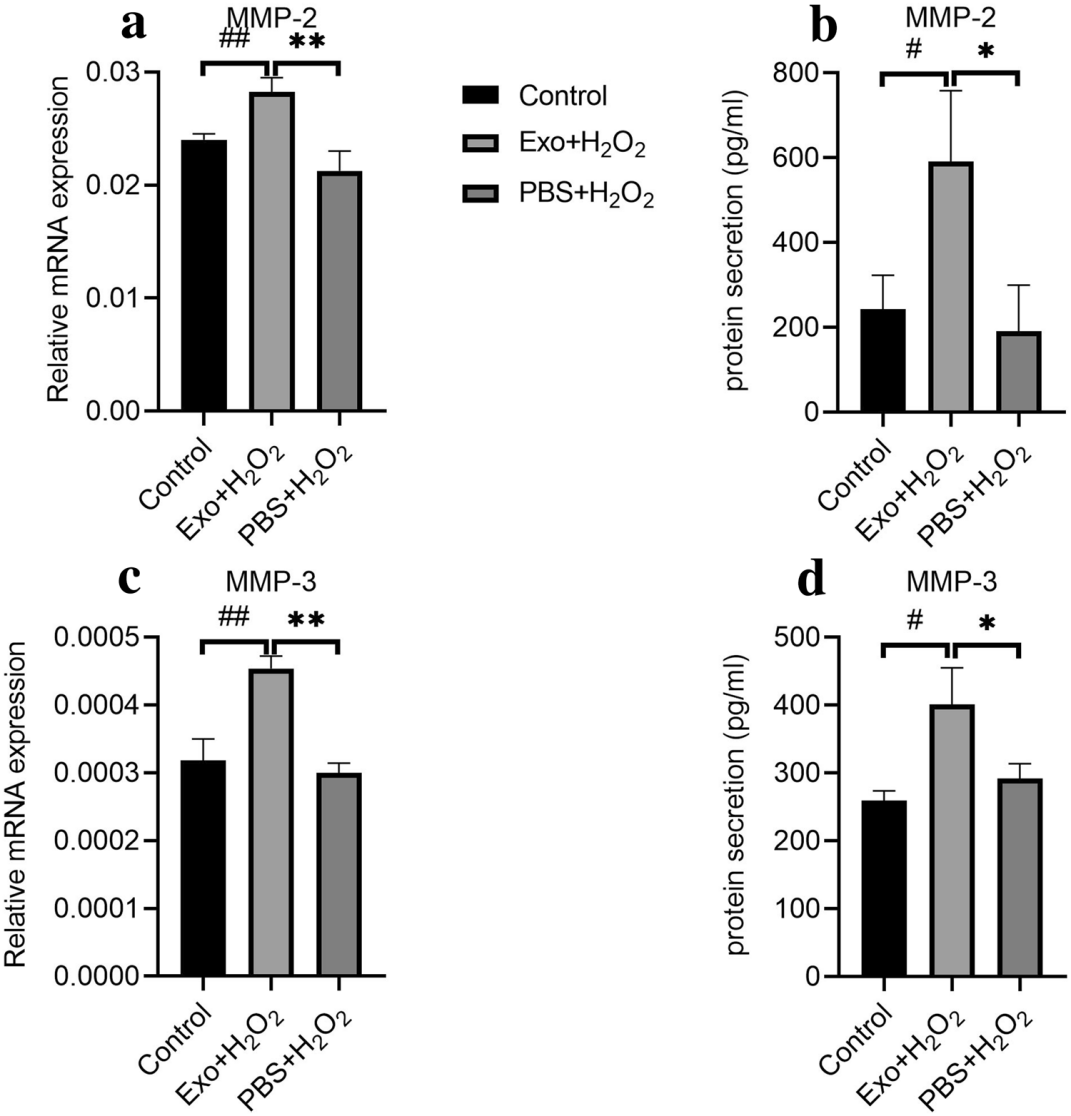
Pretreatment of hBMSC-derived exosome up-regulated the expression of matrix metalloproteinases in hTMCs when exposed to H_2_O_2_. HTMCs were pretreated with hBMSC-derived exosomes or PBS for 24 h, then exposed to H_2_O_2_ (0.1 mM) for 6 h, and a blank control group cultured with medium containing exosome-free serum. Then, hTMCs were assayed by RT-PCR to assess the mRNA expression of MMP-2 (**a**) and MMP-3 (**c**). Gene expression levels were normalized to GAPDH and presented as relative ratios. Total hTMC supernatants were analyzed for MMP-2 (**b**) and MMP-3 (**d**). n = 3 for each condition. A P value was obtained by a two-tailed unpaired t-test. *P < 0.05; **P < 0.01 compared to PBS + H_2_O_2_ group. ^#^P < 0.05; ^##^P < 0.01 compared to control. Data were presented in Supplementary Tables S4 and S5.

HTMCs were treated as described above. Expression levels of MMP-2 and MMP-3 were analyzed by RT-PCR and ELISA. After exposure to H_2_O_2_, expression levels of MMP-2 and MMP-3 were unaffected. By contrast, pretreatment with hBMSC-derived exosome up-regulated MMP-2 and MMP-3 in hTMCs exposed to H_2_O_2_ (Fig. 6).

### Effects of hBMSC-derives exoosmes on gene expression in H_2_O_2_-exposed hTMCs

**Figure 7:**
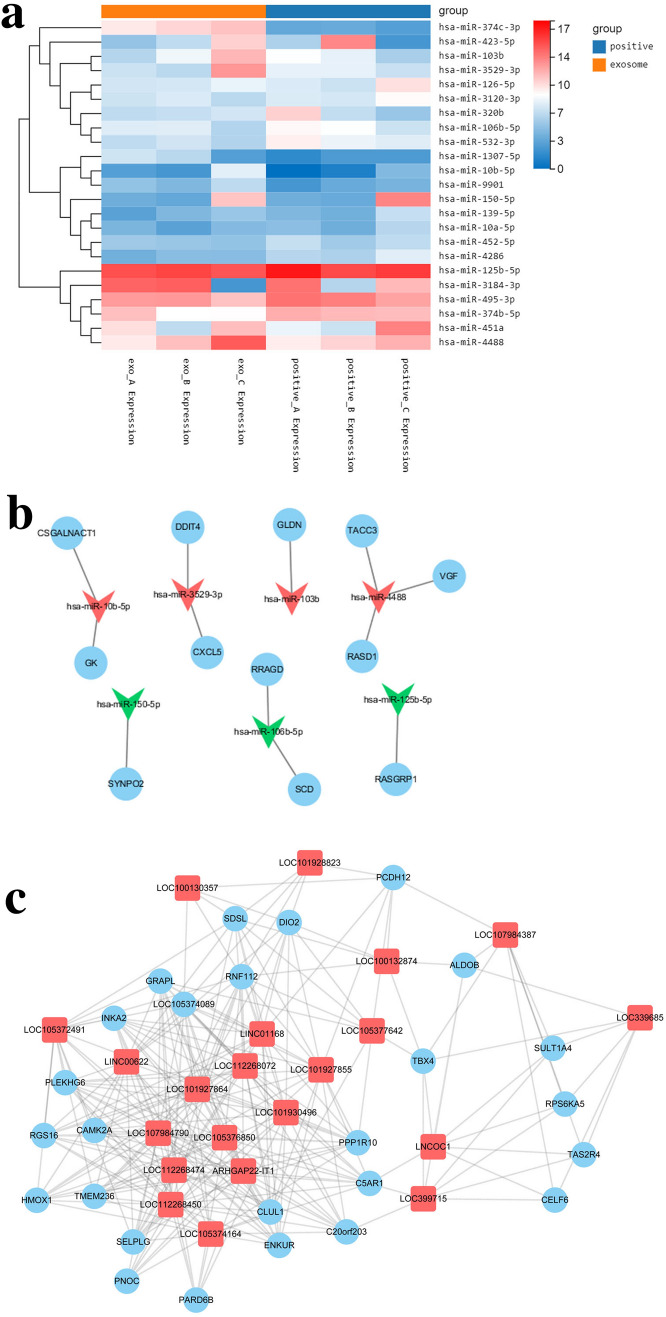
HBMSC-derived exosomes modulate gene expression of hTMCs exposed to H_2_O_2_. (**a**) Heat map of 23 DE miRNA between Exo group and control group. The horizontal axis is log2 (expression value + 1) and the vertical axis is a gene. Line indicates DE miRNA, red indicates high expression, and green indicates low expression. (**a**) was drawn using R 3.6.3 for Windows (URL: https://cran.r-project.org/bin/windows/base/old/3.6.3/R-3.6.3-win.exe). (**b**) DEmiRNA-DEmRNA regulation network. Circle indicates DE mRNA, inverted triangle indicates DE miRNA, green indicates down-regulation and red indicates up-regulation. 12 miRNA-mRNA targeting pairs were predicted in the network. (**c**) Delncrna-DEmRNA interactional subnet. Square indicates lncNRA, Circle indicates mRNA. The data of microRNA, LncRNA and mRNA were presented in Supplementary Tables S6 and S7. (**b**,**c**) were drawn using Cytoscape 3.7.2 (URL: https://github.com/cytoscape/cytoscape/releases/download/3.7.2/cytoscape-3.7.2.zip).

In this study, 6 samples were measured using BGISEQ500 platform, and an average of 11.3 GB of data was produced for each sample. Totally, 697 genes were differentially expressed (DE) through DEGseq calculation, including 23 DE miRNAs, 307 DE lncRNAs, and 367 DE mRNAs. Interestingly, expression of DE miRNA in Exo group vs. control group was identified (Fig. 7a). Moreover, 4500 target genes were identified through database queries, including 42 DE mRNAs by calculation. After further analysis of gene expression, 12 miRNA-mRNA target pairs were predicted (Fig. 7b).

Using Pearson correlation coefficient models, co-expression of DE lncRNA and DE mRNA was predicted. There were 3634 relationship pairs, including 3375 pairs of positive correlation and 259 pairs of negative correlation. DElncRNA-DEmRNA interactional network (power-law distribution R^2^ = 0.664) was constructed based on characteristics of a biological process. Notably, 9 subnets were identified using MCODE. Additionally, 21 lncRNA, 26 mRNA, and 263 pairs of interactional relations were identified in the highest score (11.435) subnet. Their levels were positively correlated and up-regulated in Exo group (Fig. 7c). Moreover, using DAVID, functional enrichment of DE mRNA was explored. GO and KEGG analysis displayed that the DE mRNAs were enriched in 138 pathways (Supplementary Table S8).

## Discussion

Detailed mechanisms underlying dysfunctional trabecular meshwork in POAG are not completely understood. However, reports^27,28^ have revealed that oxidative stress triggers trabecular meshwork dysfunction in vitro and in vivo. HTMCs under oxidative stress produce excessive iROS via mitochondria. Increased iROS production indicates oxidative stress and thus up-regulates inflammatory markers in hTMCs, including IL-1, IL-6, and IL-8. Additionally, a polymorphism in IL-1α correlated with increased IL-1 gene expression and elevated risk for POAG^29^. As a result, sustained high production of iROS and inflammatory factors are crucial features of hTMC dysfunction in POAG^30,31^. H_2_O_2_-exposed hTMCs are widely adopted in glaucoma model in vitro. In this study, survival rate of trabecular meshwork cells exposed to H_2_O_2_ was reduced, iROS was continuously produced, and IL-1α, IL-1β, IL-6, and IL-8 were up-regulated compared to control group. Besides, we successfully constructed classical glaucoma model in vitro.

Previous studies^30,31^ were confined to antioxidant effect on trabecular meshwork cells exposed to H_2_O_2_. Either sufficiency or deficiency in antioxidant supplement is not always associated with eye pathology^32^. Whereas, exosomes, as a subcellular structure containing RNAs, might affect gene expression and protect trabecular meshwork cells from oxidative stress. MSC-derived exosomes have exhibited remarkable therapeutic effects for degenerative eye diseases. For instance, MSC-derived exosomes accelerated recovery of corneal epithelium^33^. In addition, protective effects of MSC-derived exosomes on retinal ganglion cells were identified in glaucoma models^34^ . However, one study^13^ had explored potential effects of MSC-derived exosomes on trabecular meshwork, a key component of aqueous drainage channels. Herein, we focused on hTMCs, which were pretreated with hBMSC-derived exosomes, then exposed to hydrogen peroxide. Interestingly, hTMCs pretreated with hBMSC-derived exosome demonstrated enhanced survival rate, lower iROS production, and lower expression of IL-1α, IL-1β, IL-6, and IL-8 compared to those only exposed to hydrogen peroxide. These findings imply that BMSC-derived exosomes potentially alleviate dysfunction of trabecular meshwork induced by oxidative stress.

Besides up-regulated proinflammatory cytokines, iROS induced by oxidative stress potentially promotes pathophysiological changes in the outflow tract by increasing intracellular oxidative damage. Matrix metalloproteinases (MMPs), a class of zinc-containing neutral proteases implicated in regulating extracellular matrix degradation as well as interacting with microenvironment, have been proposed as a vital group of enzymes to maintain outflow tract homeostasis^35–40^. In human outflow models, MMP-2 and MMP-3 could significantly increase the outflow capacity^41^, and regulate extracellular matrix remodeling^42^. This study confirmed that cultured hTMCs secrete MMPs^43^, and detected changes in MMP-2 and MMP-3 levels upon stimulation of BMSC-derived exosome in hTMCs. Surprisingly, although oxidative stress might not influence MMPs levels in hTMCs, MMPs were up-regulated in hTMCs pretreated with hBMSC-derived exosomes. This indicates that BMSC-derived exosomes may regulate MMPs to enhance trabecular meshwork function.

To identify candidate genes responsible for alleviating oxidative stress damage in trabecular meshwork cells, gene expression was profiled in Exo and control groups. Differentially expressed genes, including 23 DE miRNAs, 307 DE lncRNAs, and 367 DE mRNAs, were identified. As shown in Fig. 7a, we analyzed the differentially expressed miRNAs in Exo and control group. Many of DE miRNAs were reported^44–47^ to be involved in the development of glaucoma, oxidative stress response, and the regulation of MMPs. For example, the expression of miR-126 in micro-vesicles was up-regulated in tears of patients diagnosed with open-angle glaucoma^46^. In our study, miR-126-5p was down-regulated in Exo group, indicating that hBMSC-derived exosomes may reduce risk of glaucoma through it. MiR-451a and miR-125b were reported^45,47^ to inhibit the expression of MMP-2. In our study, both of them were down-regulated in Exo group, indicating that hBMSC-derived exosomes up-regulated expression of MMP-2 through them.

After further analysis of gene expression, we predicted the regulatory networks of miRNA-mRNA (Fig. 7b) and lncRNA-mRNA (Fig. 7c). Many of these DE genes have been reported to be related to trabecular meshwork dysfunction and oxidative stress response. For example, in DEmiRNA-DEmRNA regulation network, miR-3529-3p was up-regulated in Exo group, while its target gene, CXCL5, was down-regulated. CXCL5, an inflammatory chemokine, was significantly elevated in aqueous humor in patients with glaucoma^48^. HBMSC-derived exosomes may reduce the inflammatory response of hTMCs under oxidative stress through the action of miR-3529-3p on CXCL5. In lncRNA-mRNA interactional subnet, DIO2 and HMOX1 act as hub nodes, co-expressing with 10 lncRNAs, respectively. DIO2 was down-regulated in Exo group. It was reported^49^ to regulate phagocytosis in trabecular network and extracellular matrix remodeling, thereby maintaining homeostasis of outflow tract. Hmox1 was up-regulated in Exo group. It is one of crucial factors in Nrf2 pathway, which plays a pivotal role in inflammation and oxidative stress response^50,51^. Several studies^50,51^ have demonstrated that up-regulated expression of Hmox1 and its metabolites have significant anti-inflammatory and antioxidant defense effects mediated by Nrf2. The expression of Hmox1 was reported^44^ to be down-regulated with increased production of iROS in trabecular meshwork. In addition, C5AR1 was down-regulated in Exo group, which was reported to be up-regulated in retinal pigment epithelium cells under oxidative stress^52^. All of the genes above may play roles in the protective effect of hBMSC-derived exosomes on trabecular meshwork under oxidative stress.

Subsequently, the results of GO and KEGG analysis demonstrated that the DE mRNAs were enriched in 138 pathways, including cell division, extracellular matrix organization, regulation of ERK1 and ERK2 cascade, etc. ERK1/2 pathway, for example, was involved in the ROS-induced cell injury^53^ and affect MMP-2 secretion in hTMCs^54^. It could be predicted that many of the 138 pathways play roles in the protective effect of hBMSC-derived exosomes on trabecular meshwork under oxidative stress.

Our study was limited to in vitro experiments on one strain of hTMCs. The conditions of hTMCs exposed to H_2_O_2_ might not entirely mirror those in POAG in vivo, however, pathological conditions may present similarity to a certain extent. Besides, hTMCs are constantly exposed to aqueous humor in vivo, while H_2_O_2_ level in human aqueous humor can reach as high as 300 μM^28^. However, this finding needs to be subjected to more strains of hTMCs or in vivo experiments. In addition, the findings from transcriptome sequencing need to be validated by a serial of experiments. Nonetheless, this study has demonstrated that hBMSC-derived exosomes could be absorbed by hTMCs, which have exerted protective effect against oxidative stress damage, and thus enabling functional preservation of hTMCs. The transcriptomic sequencing and network analysis indicate meaningful regulatory RNAs and nodes, thus providing a basis for future research on glaucoma therapy by integraing exosomes with stem cells.

## Methods and materials

### Human BMSCs culture

Human CD29^+^/CD44^+^/CD73^+^/CD90^+^/CD105^+^/CD166^+^/CD14^−^/CD31^−^/CD34^−^/CD45^−^ BMSCs were provided by Stem Cell Bank, Chinese Academy of Sciences. Cells were cultured in NutriStem MSC XF Basal Medium (Biological Industries, Israel) supplemented with 0.6% MSC XF Supplement (Biological Industries, Israel) and 1% penicillin/streptomycin. Cell cultures were maintained at 37 °C in 5% CO_2_ with the medium changed at intervals of 2 days. Cells were passaged with recombinant trypsin–EDTA solution (Biological Industries, Israel) at 80% confluence. For all experiments, hBMSCs were used at passage 2 to 5.

### Isolation and characterization of exosomes

Fresh exosome-free medium was changed at 80% confluence of hBMSCs. After 48 h, conditioned medium was collected and centrifuged at 300*g* for 10 min, 2000*g* for 10 min and 10,000*g* for 30 min, to discard the pellets and collect the supernatant each time. Thereafter, the supernatant was subjected to centrifugation in a SW 32 Ti rotor (Beckman Coulter, USA) at 100,000*g* for 70 min. After washing twice, the pellets were re-suspended in PBS. The exosome preparation was filtered through a 0.22 μm filter and stored at − 80 °C until use. At passage 2 to 5, exosomes were isolated from hBMSCs.

Exosomal morphology was characterized by transmission electron microscopy (HT7800; Hitachi, Japan). The size distribution of exosomes was measured using a ZetaView analysis system (PMX 110; Particle Metrix, Germany). Classic exosomal surface markers were examined by Western blotting analysis. Total protein from exosomes was extracted in lysis buffer, and a protein BCA assay kit was used to measure concentration. Lysates were separated on 10% SDS–polyacrylamide gel and proteins were transferred to polyvinylidene difluoride membranes. Then, membranes were incubated with 5% milk for 1 h and probed overnight at 4 °C with antibodies targeting HSP70 (ab181606, 1:1000 dilution, Abcam, USA) and CD9 (ab92726, 1:2000 dilution, Abcam, USA). After washing, blots were incubated with peroxidase-conjugated anti-rabbit IgG horseradish and peroxidase secondary antibodies (ab205718, 1:2000 dilution, Abcam, USA) at room temperature for 1 h. The Alpha FluorChem E system (ProteinSimple, USA) was used to visualize the protein bands.

### Human trabecular meshwork cells culture and characterization

A human normal trabecular meshwork cell line was obtained from ScienCell Research Labs (Carlsbad, CA, USA). HTMCs were cultured in DMEM/Ham’s F12 (M&C gene technology, Beijing, China) supplemented with penicillin/streptomycin and 10% fetal bovine serum (FBS). Cell cultures were maintained at 37 °C in 5% CO_2_ with medium being changed at intervals of 3 days and cells passaged with 0.05% trypsin/EDTA at 80% confluence. HTMCs were characterized through Dexamethasone treatment. HTMCs were exposed to dexamethasone (Dex, 100 nmol; Sigma-Aldrich, USA) or vehicle (0.1% ethanol; Sigma-Aldrich, USA) for 48 h. Then the gene expression of myocilin was detected by RT-PCR (Supplementary Fig. S1a). And protein expression of myocilin in cell lysate was detected by ELISA (Supplementary Fig. S1b). Data were presented in Supplementary Table S1. For all experiments, hTMCs were used at passage 2 to 5.

### Exosome tracking

HBMSC-derived exosomes were stained with a green fluorescent dye (PKH67, Sigma-Aldrich, USA). Briefly, 200 μl exosome suspension with 1.0 × 10^8^ exosomes were mixed with equal volume dilution containing 2 μl dye and incubated at 37 °C for 5 min in dark. An equal volume of 10% BSA-PBA solution was added to stop dyeing. Then, they were subjected to ultracentrifugation at 100,000*g* for 70 min. The pellets were re-suspended in 100 μL PBS. HTMCs were cultured as described above. At 60% confluence, the cells were exposed to the stained exosomes or dye for 0, 6, 12, and 24 h respectively. Then, the hTMCs were stained with phalloidin (Sigma-Aldrich, USA) and 4, 6-diamidino-2-phenylindole (DAPI, Solarbio Science & Technology, Beijing, China) sequentially, and mounted on a slide and observed under a fluorescence microscope.

### Oxidative stress and human BMSC-derived exosomes pretreatment

HTMCs were seeded at a density of 1 × 10^6^ cells per well then cultured in normal growth medium for 24 h. After washing 3 times with PBS, the cells were cultured in a complete media (containing exosome-free serum) with hBMSC-derived exosomes (1 × 10^8^ particles/ml) or PBS for 24 h. After another 3-time-washing, hTMCs were treated with 0.1 mM H_2_O_2_ for 6, 12, and 24 h respectively. Then, the supernatant and cell lysis solution were collected for further analysis.

### Cell counting kit-8

After treatments, cell viability was quantified at 6, 12, and 24 h using a cell counting kit-8 (Sigma-Aldrich, USA). A total of 100 ml aliquots of medium was transferred to 96 well plates and absorbance at 450 nm was measured using a spectrophotometer (Bio-rad; USA) and normalized to control.

### Measurement of intracellular ROS

Intracellular ROS (iROS) was determined by 2′,7′-dichlorodihydrofluorescein diacetate (DCFDA; Sigama, USA). After the above treatments, the culture medium with H_2_O_2_ was discarded and hTMCs were incubated with 10 μM of DCFDA for 30 min in PBS. This was followed by PBS wash, incubation in media for 20 min, and trypsinization. Lastly, cells were collected in PBS and kept on ice until analyzed by FACS Aria III flow cytometer (Becton, Dickinson and Company; USA). An average of 10,000 cells was analyzed in each experiment. All experiments were performed in triplicate.

### Real-time PCR (RT-PCR)

Total RNA was extracted from cultured cells using Trizol reagent (TaKaRa, Japan) as per the manufacturer's protocol. After quantifying the RNA concentration, a cDNA library was constructed using a Reverse Transcriptase kit (TaKaRa, Japan) following to the manufacturer's instructions. RT-PCR was performed on a RT PCR system (7500; ABI, USA) using BeyoFast SYBR Green qPCR Mix (Beyotime, China). Samples were normalized to internal control GAPDH. Primer sequences are listed as follows: IL-1α (forward primers 5′-AGG CTG CAT GGA TCA ATC TGT GTC-3′; reverse primers 5′-CTT CCT CTG AGT CAT TGG CGA TGG-3′), IL-1β (forward primers 5′-CTG AAA GCT CTC CAC CTC CA-3′; reverse primers 5′-TCA TCT TTC AAC ACG CAG GA-3′), IL-6 (forward primers 5′-GGT GTT GCC TGC TGC CTT CC-3′; reverse primers 5′-AGA TGC CGT CGA GGA TGT ACC G-3′), IL-8 (forward primers 5′-TCT CTT GGC AGC CTT CCT GA-3′; reverse primers 5′-TTT CTG TGT TGG CGC AGT GT-3′), MMP-2 (forward primers 5′-GCC TCT CCT GAC ATT GAC CTT GG-3′; reverse primers 5′-CAC CAC GGA TCT GAG CGA TGC-3′), MMP-3 (forward primers 5′-GCC AGG GAT TAA TGG AGA TG-3′; reverse primers 5′-ATT TCA TGA GCA GCA ACG AG-3′), and GAPDH (forward primers 5′-TCG ACA GTC AGC CGC ATC TTC TTT-3′; reverse primers 5′-ACC AAA TCC GTT GAC TCC GAC CTT-3′).

### ELISA measurement

The levels of IL-1α, IL-1β, IL-6, IL-8, MMP-2, and MMP-3 in cell culture supernatant were detected by a multi-detection microplate reader using a double-antibody sandwich ELISA kit (Boster Biological Technology, USA). All results were normalized against a standard curve.

### Sequencing of miRNA and identification of DE miRNA

HTMCs in Exo group (Exo A, B and C) were pretreated with hBMSC-derived exosomes for 24 h, then exposed to 0.1 mM H_2_O_2_ for 6 h. HTMCs in control group (control A, B and C) were pretreated with PBS for 24 h, also followed by exposure to 0.1 mM H_2_O_2_ for 6 h. Total RNA from two groups was collected using Trizol reagent (TaKaRa, Japan) according to the manufacturer's protocol, then qualified and quantified using a Nano Drop and Agilent 2100 bioanalyzer (Thermo Fisher Scientific, USA). TruSeq Small RNA Library Prep Kit (Illumina, USA) was used to construct the cDNA libraries. According to the manufacturer’s instructions, a 5′ adapter and a 3′ adapter were ligated to the small RNA molecules using T4 RNA ligase. Subsequently, the adapter-ligated small RNAs were transcribed into cDNA by SuperScript II Reverse Transcriptase (Invitrogen, USA). After several rounds of PCR amplification, the cDNA fragments were enriched. The products were selected by agarose gel electrophoresis and purified by QIAquick Gel Extraction Kit (QIAGEN, CA). The Agilent 2100 bioanalyzer was used to check the fragments size distribution. The final ligation PCR products were sequenced using the BGISEQ-500 platform (BGI-Shenzhen, China).

To obtain clean reads, small RNA sequencing data were filtered by SOAPnuke^55^ to removes reads based on the following criteria: low-quality, less than 18nt, with 5′ adapter contamination or poly A, or without 3′ adapter sequence or insert fragment. Bowtie2^56^ was used to compare the clean reads with reference sequence. The expression level of miRNAs was measured by transcripts per million (TPM). The differentially expressed miRNAs were detected using DEGseq^57^ with the standard of false discovery rate (FDR) ≤ 0.001, and fold change ≥ 2 or fold change ≤ 0.5.

### Sequencing of lncRNA and mRNA and identification of DE lncRNA and DE mRNA

Total RNA was extracted as described above, then qualified and quantified using a Nano Drop and Agilent 2100 bioanalyzer. TruSeq Stranded Total RNA Kit (Illumina, USA) was used to construct the cDNA libraries. After removal of RNA (rRNA), the RNA was fragmented into small pieces. The RNA fragments were used to synthesize first strand cDNA with random primers. In the process of second strand cDNA synthesis, dUTP replaced dTTP. After the addition of a single 'A' base, ligation of the adapter and UDG (uracil-DNA glycosylase) treatment, the products are enriched with PCR and final cDNA library was created. The Agilent 2100 bioanalyzer was used to check the fragments size distribution. The final ligation PCR products were sequenced using the BGISEQ-500 platform (BGI-Shenzhen, China).

RNA sequencing data were filtered by SOAPnuke to eliminate reads containing adapter, unknown base N ratio greater than 5%, or ratio of bases with mass value less than 10 greater than 20%. After obtaining clean reads, HISAT^58^ was used to compare the clean reads with reference genome sequence. RSEM^59^ was used to calculate gene expression, while FPKM was used to standardize gene expression. The differentially expressed mRNAs and lncRNAs were detected using DEGseq with with the standard of false discovery rate (FDR) ≤ 0.001, and fold change ≥ 2 or fold change ≤ 0.5.

### Bioinformatics analysis

According to the results of differentially expressed genes, hierarchical clustering analysis was performed using PheatMap function in R software. The co-expression relation pair was selected by Pearson's correlation coefficient with the standard of P value < 0.01 and |cor| > 0.9. The target genes of miRNA were quested through the miRTarBase^60^ database. The network graph was drawn using Cytoscape software, while the subnets were mined by MCODE in Cytoscape. David (the Database for Annotation, Visualization and Integration Discovery, https://david.ncifcrf.gov/summary.jsp) was used for enrichment analysis of KEGG^61–63^ pathway and GO biological process.

### Statistical analysis

All experiments were conducted at least 3 biological replicates. Results were presented as mean ± standard deviation (SD) of three independent experiments. A P value for difference was determined by t-test using GraphPad Prism software 9 (URL: https://www.graphpad.com/) and Microsoft Excel. A differences was considered significant when a P value were less than 0.05 (P < 0.05).

## Supplementary Information

Supplementary Information 1.

Supplementary Information 2.

Supplementary Information 3.

Supplementary Information 4.

## Data Availability

Supplementary information accompanies this paper.
