# Supplementary material for: Mesenchymal stem cell-derived exosomes protect trabecular meshwork from oxidative stress
Source: Sci Rep. 2021 Jul 21;11:14863. doi: 10.1038/s41598-021-94365-4 (PMC8295363; doi:10.1038/s41598-021-94365-4)

Supplementary Materials for

**Mesenchymal Stem Cell-Derived Exosomes Protect** **Trabecular Meshwork from** **Oxidative Stress**

Ying-chao Li^1,2^, Juan Zheng^3^, Xi-zi Wang^3^, Xin Wang^4^, Wen-jing Liu^2^, and Jian-lu Gao^1,4^*

^1^Department of Ophthalmology, Liaocheng People's Hospital, Cheeloo College of Medicine, Shandong University

^2^Department of Ophthalmology, Taian City Central Hospital

^3^Joint Laboratory for Translational Medicine Research, Beijing Institute of Genomics, Chinese Academy of Sciences & Liaocheng People’s Hospital

^4^Department of Ophthalmology, Liaocheng People's Hospital

*Corresponding author. Department of Ophthalmology, Liaocheng People's Hospital, Cheeloo College of Medicine, Shandong University, Liaocheng, Shandong 252000, China. E-mail: drgaojianlu@163.com. Tel: +8618663500588


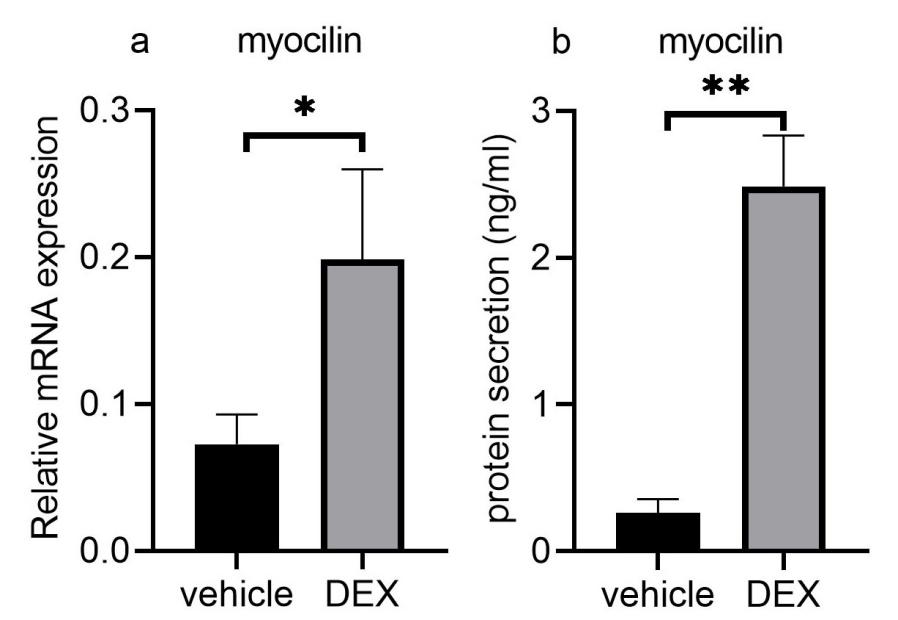


Figure S1. Characterization of hTMCs through dexamethasone treatment. HTMCs were exposed to dexamethasone or vehicle for 48 hours. The gene expression of myocilin was detected by RT-PCR (a). Protein expression of myocilin in cell lysate was detected by ELISA (b). n=3 for each condition. A P value was obtained by a two-tailed unpaired t-test. *P <0.05; **P < 0.01.

Table S1. Data of gene and protein expression of myocilin in hTMCs exposed to dexamethasone or vehicle.

|  | vehicle | DEX |
| --- | --- | --- |
| PCR | 0.049655 | 0.128669 |
|  | 0.080054 | 0.241745 |
|  | 0.088162 | 0.225604 |
| ELISA | 0.18588 | 2.475416 |
|  | 0.229352 | 2.142129 |
|  | 0.367014 | 2.837685 |
|  |  |  |

Table S2. Cell viability of three groups for 6, 12, and 24 hours. (Exo+H_2_O_2_ group: hTMCs were pretreated with hBMSC-derived exosomes for 24hours, then exposed to 0.1mM H_2_O_2_; PBS+H_2_O_2_ group: hTMCs were pretreated with PBS for 24hours, then exposed to 0.1mM H_2_O_2_; Control group: hTMCs were culture in DMEM-Ham’s F12 for 24hours，then changed for new medium.)

|  | Control | Exo+H_2_O_2_ | PBS+H_2_O_2_ | Exo+H_2_O_2_：Control | PBS+H_2_O_2_：Control |
| --- | --- | --- | --- | --- | --- |
| 6h | 1.042 | 0.989 | 0.687 | 94.91% | 65.93% |
|  | 0.999 | 1.001 | 0.62 | 100.20% | 62.06% |
|  | 1.014 | 0.989 | 0.598 | 97.53% | 58.97% |
| 12h | 1.132 | 1.156 | 0.697 | 102.12% | 61.57% |
|  | 1.101 | 0.91 | 0.648 | 82.65% | 58.86% |
|  | 1.305 | 1.041 | 0.716 | 79.77% | 54.87% |
| 24h | 1.202 | 0.822 | 0.407 | 68.39% | 33.86% |
|  | 1.899 | 0.848 | 0.434 | 44.66% | 22.85% |
|  | 1.39 | 0.851 | 0.418 | 61.22% | 30.07% |

Table S3. Data of the differentially iROS between Exo+H_2_O_2_ group and PBS+H_2_O_2_ group

| Control | Exo+H_2_O_2_ | PBS+H_2_O_2_ |
| --- | --- | --- |
| 131 | 375 | 841 |
| 198 | 475 | 1561 |
| 261 | 552 | 1210 |
|  |  |  |

Table S4. Data of the differentially mRNA expression of inflammatory factors and MMPs between Exo+H_2_O_2_ group and PBS+H_2_O_2_ group

|  | Control | Exo+H_2_O_2_ | PBS+H_2_O_2_ |
| --- | --- | --- | --- |
| IL-1alpha | 0.000934 | 0.001353 | 0.002763 |
|  | 0.00094 | 0.001409 | 0.003027 |
|  | 0.000974 | 0.001337 | 0.003152 |
| IL-1beta | 0.008483 | 0.011007 | 0.01388 |
|  | 0.009806 | 0.012065 | 0.016504 |
|  | 0.00776 | 0.013089 | 0.015898 |
| IL-6 | 0.021437 | 0.031635 | 0.034917 |
|  | 0.02269 | 0.028596 | 0.036001 |
|  | 0.024785 | 0.030501 | 0.034584 |
| IL-8 | 0.002542 | 0.001979 | 0.010425 |
|  | 0.002532 | 0.003926 | 0.008551 |
|  | 0.00211 | 0.00416 | 0.008176 |
| MMP-2 | 0.023799 | 0.029591 | 0.021832 |
|  | 0.023615 | 0.027161 | 0.022673 |
|  | 0.024618 | 0.028072 | 0.019317 |
| MMP-3 | 0.000328 | 0.000475 | 0.000296 |
|  | 0.000344 | 0.000447 | 0.000316 |
|  | 0.000283 | 0.000441 | 0.000287 |
|  |  |  |  |

Table S5. Data of the differentially protein expression of inflammatory factors and MMPs between Exo+H_2_O_2_ group and PBS+H_2_O_2_ group

|  | Control | Exo+H_2_O_2_ | PBS+H_2_O_2_ |
| --- | --- | --- | --- |
| IL-1alpha | 3.985725 | 4.694218 | 14.09772 |
|  | 2.575105 | 8.268512 | 21.19629 |
|  | 1.872978 | 6.832428 | 12.9944 |
| IL-1beta | 1.41616 | 3.85048 | 12.89224 |
|  | 1.0684 | 5.24152 | 10.45792 |
|  | 0.37288 | 9.7624 | 12.19672 |
| IL-6 | 121.1135 | 225.2687 | 1053.498 |
|  | 122.2274 | 249.7758 | 982.7615 |
|  | 148.9625 | 238.6362 | 1318.063 |
| IL-8 | 62.75 | 155.49 | 487.55 |
|  | 77.4 | 117.76 | 544.9 |
|  | 102.34 | 157.08 | 539.57 |
| MMP-2 | 156.452 | 469.154 | 104.335 |
|  | 312.803 | 781.856 | 156.452 |
|  | 260.686 | 521.271 | 312.803 |
| MMP-3 | 267.77 | 351.23 | 267.39 |
|  | 242.96 | 392.58 | 300.47 |
|  | 267.77 | 458.74 | 308.74 |
|  |  |  |  |

Table S6. List of microRNA expression of Exo group (hTMCs pretreated by hBMSC-derived exosomes and followed by exposure to H_2_O_2_) and control group (hTMCs pretreated by PBS and followed by exposure to H_2_O_2_)

Table S7. List of LncRNA and mRNA expression of Exo and control group

Table S8. Results of DE mRNA KEGG pathway and GO term annotation

Fig. 1b


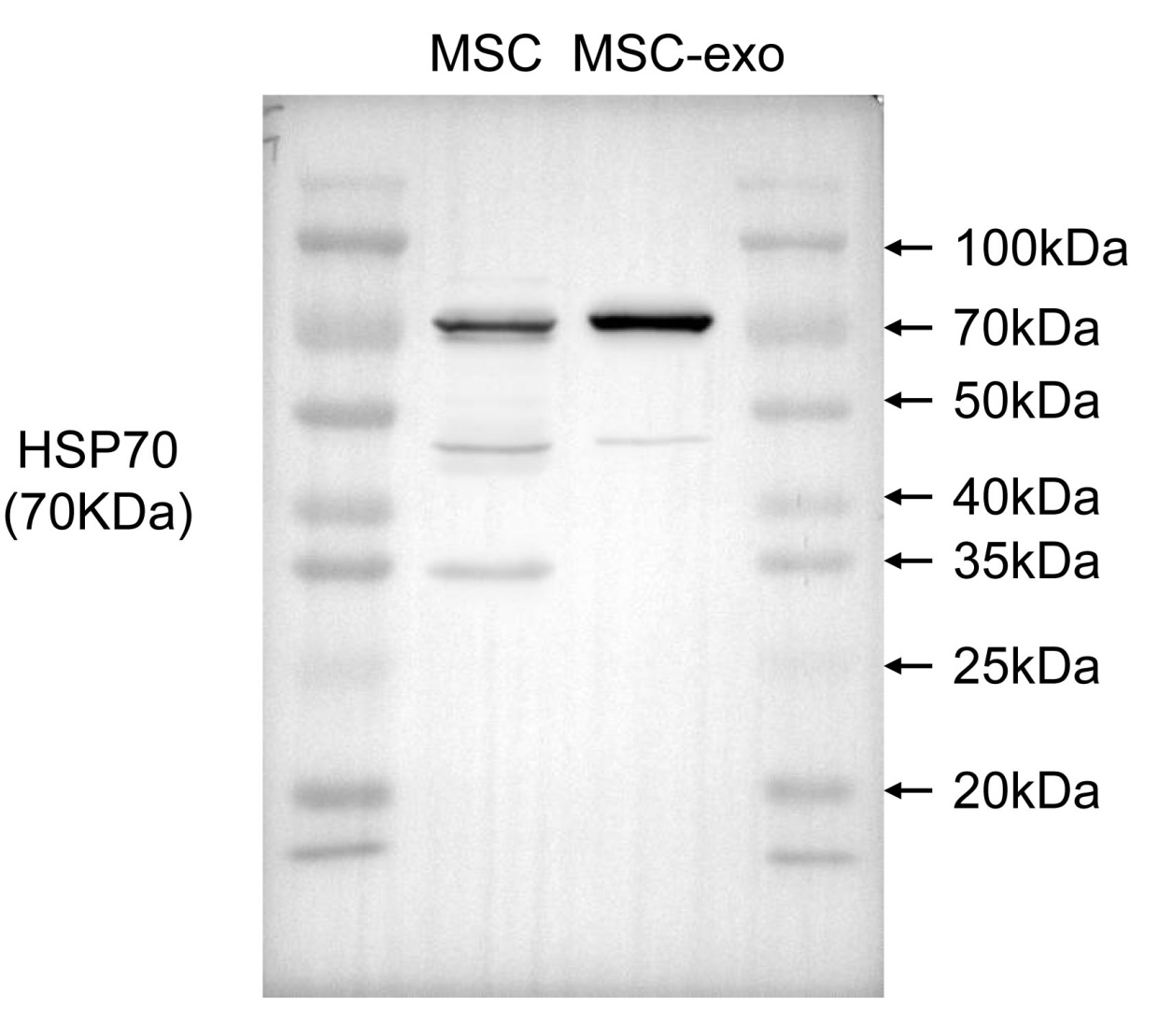


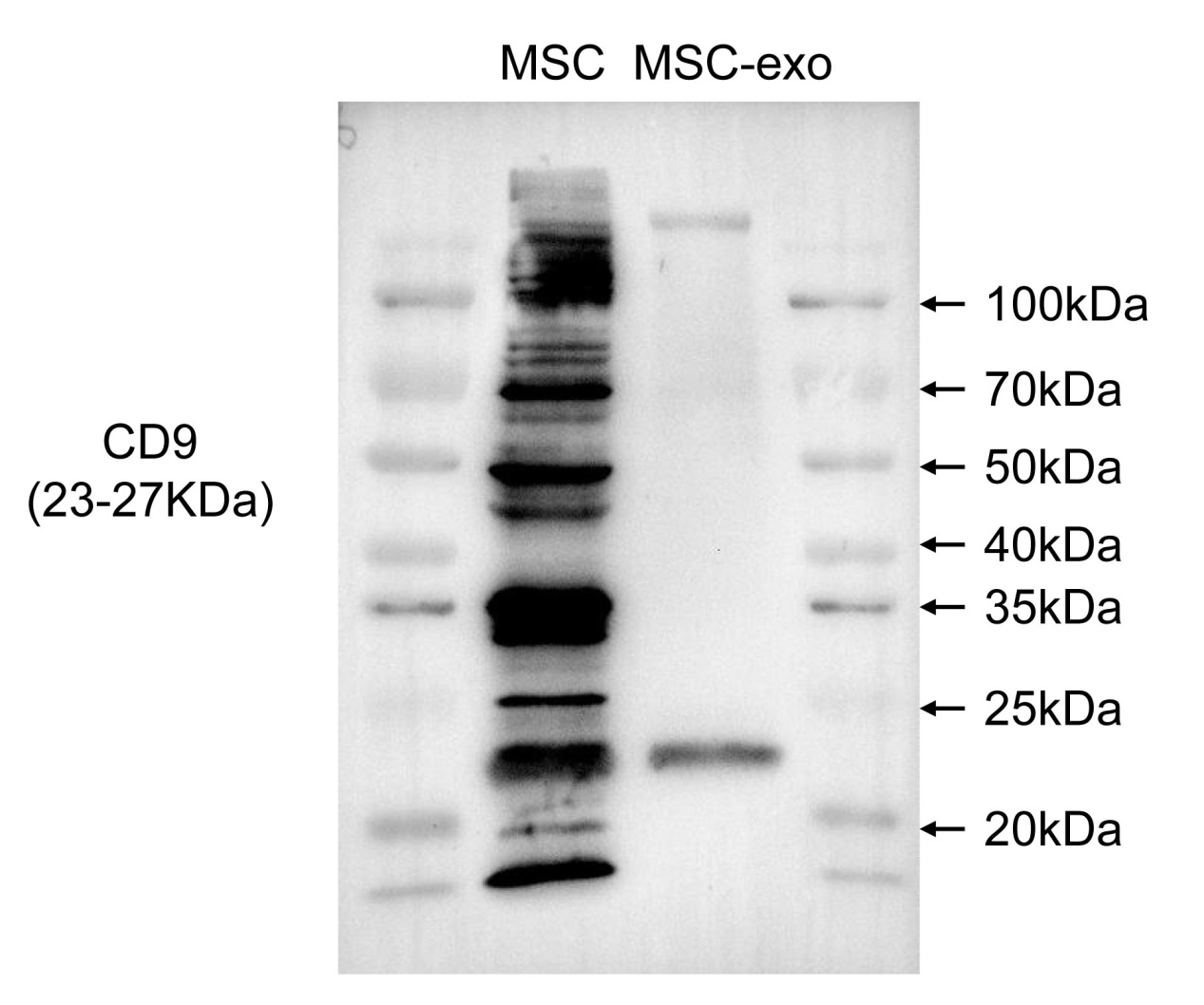

Supplement: Supplementary file 1 — Supplementary Information 1. [file 41598_2021_94365_MOESM1_ESM.docx]
